# Imaging of the vascular distribution of the outer ear using optical coherence tomography angiography for highly accurate positioning of a hearable sensor

**DOI:** 10.1063/5.0203582

**Published:** 2024-05-23

**Authors:** Juyeon Hong, Daewoon Seong, Dongwan Kang, Hyunmo Kim, Jeong Hun Jang, Mansik Jeon, Jeehyun Kim

**Affiliations:** 1 School of Electronic and Electrical Engineering, College of IT Engineering, Kyungpook National University, 80, Daehak-ro, Buk-gu, Daegu 41566, South Korea; 2 Department of Otolaryngology, School of Medicine, Ajou University, 206, World cup-ro, Yeongtong-gu, Suwon 16499, South Korea

## Abstract

Novel hearable technology is securely and comfortably positioned within the ear canal minimizing inaccuracies caused by accessory movements during activities. Despite extensive research on hearable technologies within the outer ear, there is a lack of research in the field of vascular imaging and quantitative analysis in the outer ear *in vivo*, which is one of the crucial factors to select the appropriate sensor position. Therefore, in this paper, we introduced optical coherence tomography angiography (OCTA)-based qualitative and quantitative analyses to visualize the inner vasculature of the outer ear to acquire vascular maps for microvascular assessments *in vivo*. By generating maximum amplitude projection images from three-dimensional blood vascular volume, we identified variations of blood vessel signal caused by the different biological characteristics and curvature of the ear among individuals. The performance of micro-vascular mapping using the proposed method was validated through the comparison and analysis of individual vascular parameters using extracted 20 vascular-related variables. In addition, we extracted pulsatile blood flow signals, demonstrating its potential to provide photoplethysmographic signals and ear blood maps simultaneously. Therefore, our proposed OCTA-based method for ear vascular mapping successfully provides quantitative information about ear vasculature, which is potentially used for determining the position of system-on-chip sensors for health monitoring in hearable devices.

## INTRODUCTION

Physiological parameter trackers are an evolving technology that can monitor and collect physiological data, such as heart rate (HR), temperature (T), and oxygen saturation (SpO_2_).^1^ Heart rate and oxygen saturation are the most indispensable signals that reflect the health condition of the human body.^2,3^ These physiological parameters can be estimated using devices that rely on photoplethysmography (PPG), a technology that measures the absorption and reflection of light in the vascular bed in various peripheral body regions.^4^ The popularity of activity trackers is evident in their widespread usage in today's society and the ongoing evolution of tracker models used to improve accuracy and convenience for end users.^5^ In addition to individual consumer use, several healthcare companies have incorporated wearable technology to encourage physical activity to reduce healthcare costs and the burden of chronic disease.^6^ High-speed data transmission in conjunction with the growth of mobile networks and miniaturized microprocessors have enabled the development of wearable technology.^7^ Wearable technology, often referred to as wearables, represents a category of electronic devices designed to be worn as integrated accessories within clothing, with smartwatches and fitness trackers standing out as exemplars.^8^ Wearable technology has the potential to revolutionize healthcare and medical research by enabling accessible, continuous, and longitudinal health monitoring.^9^

Wearables, especially wrist-worn devices, have some limitations to overcome. Accurate measurement of PPG values depends on the degree of contact of the sensor and the distribution of subcutaneous blood vessels. Motion artifacts caused by displacement of the PPG sensor over the skin due to physical activity or periodic wrist movements will cause measurement errors.^10^ The precision of wrist-based devices is highest during periods of rest and diminishes during physical exercise.^11^ In addition, the accuracy of the wearable is also affected by peripheral blood perfusion. To address these limitations, a proposed solution involves the placement of the sensor in areas with increased perfusion.^12^ Among the alternative measurement regions, the ear canal has been suggested as a promising measurement site for physiological parameters that can potentially combine minimal invasiveness and wearability with reliable and accurate recordings in a variety of settings.^1^ Because the main vasculature is located in superficial regions close to the skin, it can provide better signal quality and enhanced stability. This area has sufficient blood flow for high-quality PPG signals and reliable pulse rate (PR) and SpO_2_ monitoring.^13^ This site can also offer excellent fixation and unobtrusiveness, thus facilitating long-term monitoring in everyday living conditions and protecting against adverse environmental conditions for field applications in demanding conditions.^14^ The absence of bone and primarily cartilage and blood damage suggests that the ear is suitable for continuous HR monitoring using PPG.^15^ The external ear is supplied by arteries that supply blood to the brain and remains adequately perfused during low-perfusion conditions. Additionally, the anatomy of the external ear canal would provide a natural anchoring for the sensor. The concha area connected to the ear canal comprises a relatively flat space and a rich distribution of blood vessels; thus, it may be suitable for the positioning of the sensor.

Therefore, the new wearable technology (known as “hearable”) is safely and comfortably placed inside the ear canal, thus reducing the rate of incorrect accessory movements recorded during activities. The usefulness and differentiation of hearables (wireless electronic products worn in, on, or around the ear) can be secured as they can overcome the disadvantages of wearables.^16^ Therefore, it is required to measure quantitatively the blood vessel map of the ear to enhance the effectiveness of hearables.

Considering that system-on-chip (SoC) sensors are intended to measure selectively HR and oxygen saturation in microvessels, it is deemed appropriate to position these in areas with the highest microvessel density. Accurate sensor measurements are essential for successful analysis of internal blood vessels. Therefore, it is crucial to place the sensors near adjacent microvessels. However, owing to restrictions associated with capillary-level vascular information *in vivo* using existing methods, most studies have been conducted *ex vivo* on cadavers.^17^ Consequently, there have been limitations in creating high-precision hearable devices based on actual vascular distribution information. Since the hearable device needs to be attached to alive individuals, it is essential to demonstrate the feasibility of measuring blood flow signals *in vivo* based on actual vascular distribution information. In addition, while various research studies have been actively pursued in hearable-related fields, such as temperature measurements for SoC sensor localization in the external ear,^18–20^ HR and pulse rate,^21–23^ and peripheral oxygen saturation,^12,24,25^ there has been a lack of research on vascular imaging and quantitative analyses. Therefore, there is a need for equipment that can evaluate the distribution of microvessels with high precision in limited *in vivo* locations.

Optical coherence tomography (OCT) is an interferometer-based optical imaging technique, which provides noninvasive internal structural information with high-resolution in real-time.^26^ OCT has been extensively utilized in various fields, such as ophthalmology,^27,28^ otolaryngology,^29,30^ dentistry,^31,32^ and industrial applications.^33,34^ Specifically, in terms of microvasculature imaging, optical coherence tomography angiography (OCTA) was introduced and was extensively utilized in a diverse range of applications.^35,36^ OCTA is specialized for vasculature imaging and does not require hardware modifications to conventional OCT systems. OCTA stands out for its ability to assess microvascular structures, offering a noninvasive, label-free, and depth-resolved portrayal of microvasculature with unparalleled capillary-level precision.^37^ OCTA images were derived from the diverse backscattering of light originating from vascular and neural sensory tissues. The intensity and phase of backscattered light vary with the inherent motion of tissues, inherently making OCTA images as dynamic contrast images that allow measurements of intravascular red blood-cell movements.^38^ In addition, by continuously tracking the OCTA signal variation in microvessel areas, the PPG waveform can be obtained.^39^ Therefore, the OCTA technique is suitable to obtain simultaneously high-resolution microvessel maps and PPG waveforms, which is one of the important factors measured by conventional SoC sensors.

In this study, we propose OCTA-based, noninvasive microvasculature map imaging of the ear. To stably obtain the OCTA-based vessel signals, we customized a probe tip for ear imaging and integrated it with the original sample probe. In addition, we selected three representative regions of the ear and imaged 10 healthy volunteers. The cross-sectional images, including structural and vascular images, were shown to verify vessel location in the depth direction. In addition, maximum amplitude projection (MAP) images were extracted and employed for qualitative comparisons of all the regions and volunteers. Moreover, quantitative vascular analyses were also conducted based on 20 vascular-related, factors. Furthermore, we measured the PPG waveform by detecting blood volume differences in the vessel regions. Based on these results, the proposed OCTA-based, ear vascular mapping method has the potential to be utilized in the determination of the location of hearable sensors by providing comprehensive vascular information at the microscale. Our research marks the first endeavor to utilize OCTA for *in vivo* imaging in order to investigate sensor positioning for hearable devices. The successful outcomes of our study have the potential to serve as a model for other research endeavors requiring *in vivo* analysis of ear vascular distribution.

## RESULTS

### Qualitative analysis of vascular distribution at each position

**FIG. 1.**
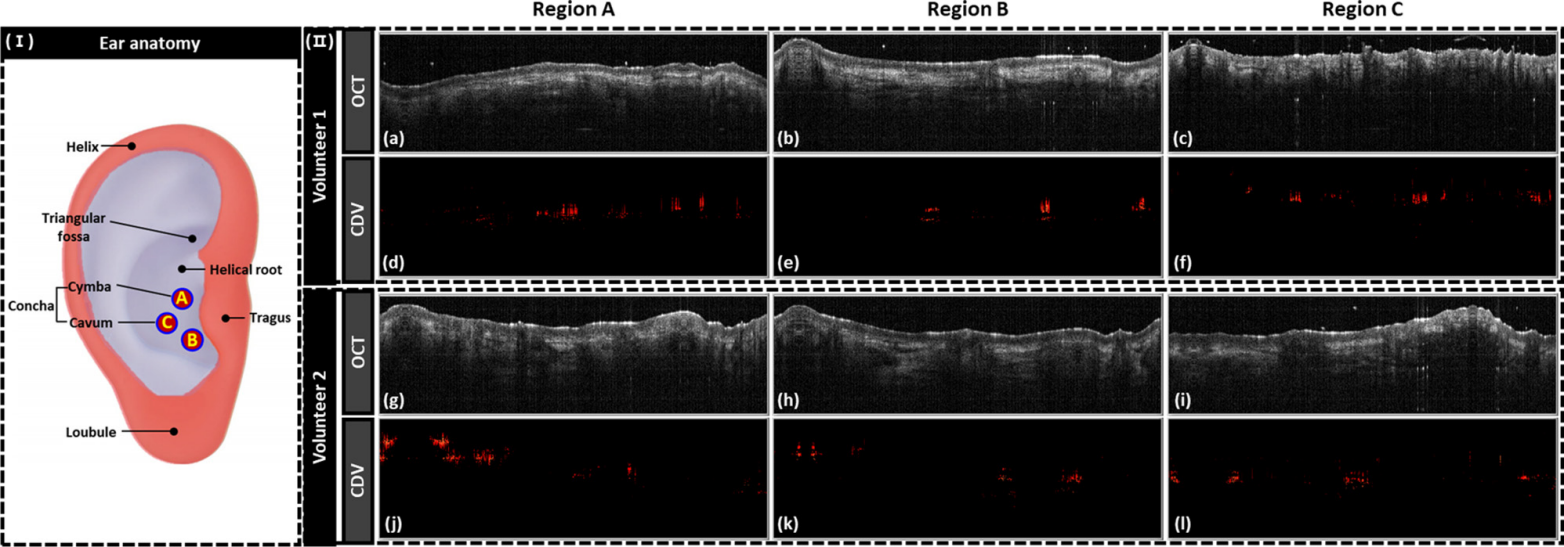
(I) Vascular distribution region within the external ear and imaging locations A, B, and C. The branching regions of the superior temporal (red) and posterior auricular arteries (blue). (II) (a)–(c) cross-sectional images of volunteer 1. (d)–(f) CDV images from volunteer 1. (g)–(i) Cross-sectional images from volunteer 2. (j)–(l) CDV images from volunteer 2.

Figure 1 shows the representative cross-sectional images from two volunteers, as well as the images containing vascular information obtained by the CDV algorithm for regions A, B, and C. In terms of the morphological cross-sectional images [Figs. 1(II-a)–1(I-c) and 1(II-g)–1(II-i)], the distinctive features of each subsurface (which were distinguished) were the epidermis layer, dermis layer, and hair follicles. Therefore, the intensity-based morphological images also possess the capacity to provide biological features of the SoC target location at high resolution and in real time. Moreover, through the customized software with the CDV algorithm, the vascular signal of each position can be extracted and mapped as shown in Figs. 1(II-d)–1(II-f) and 1(II-j)–1(II-l). As vascular information is obtained from cross-sectional data (i.e., depth-direction data), it is possible to obtain not only the blood vessel signal but also located depth of the vessel.

**FIG. 2.**
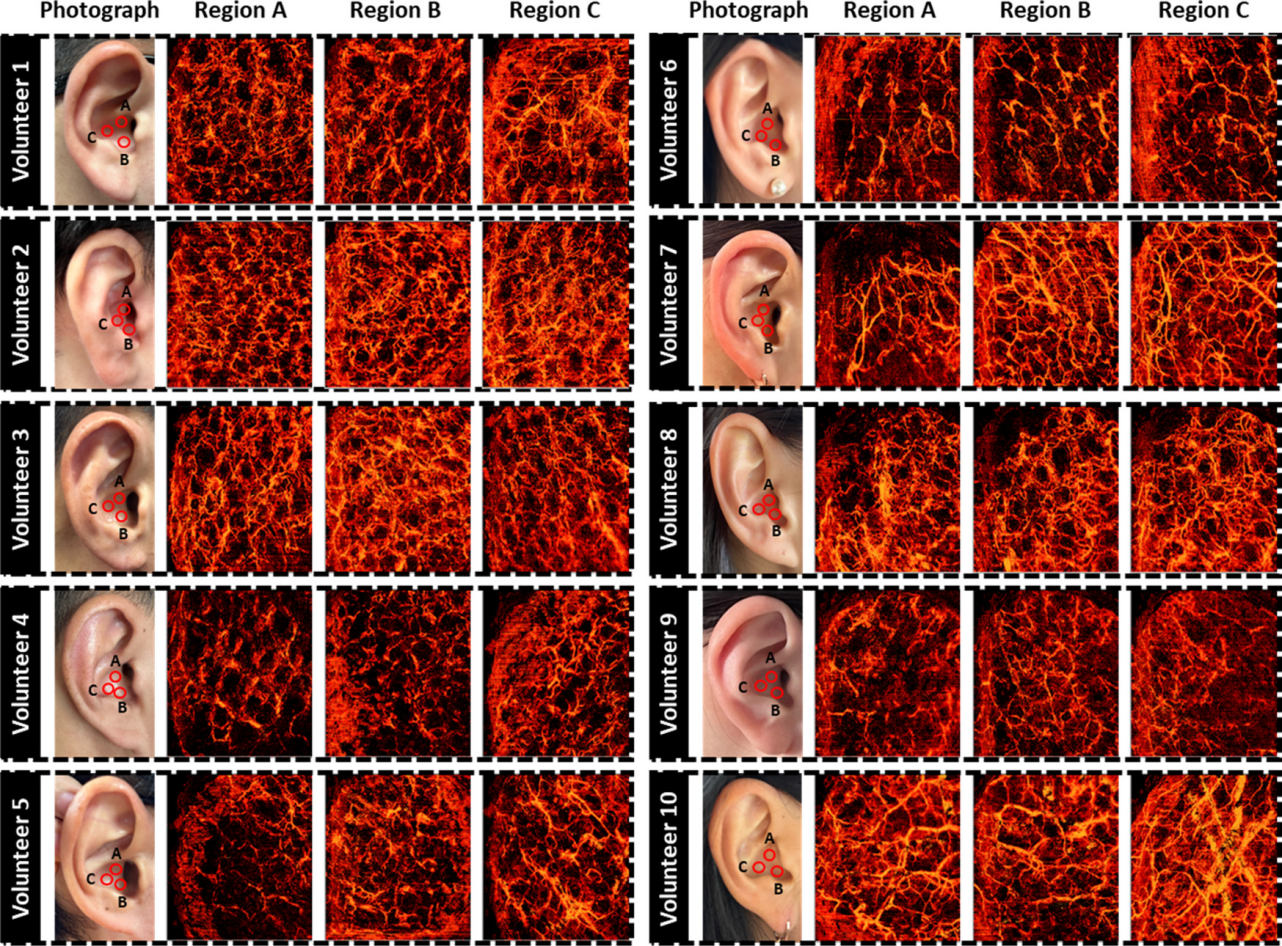
Acquired vascular distribution maps of regions A, B, and C of the volunteers.

In addition to the cross-sectional image-based analysis, we combined the vascular signals obtained from each cross-sectional image and generated MAP images of the entire 3D volume to obtain a comprehensive vascular map. The obtained vascular maps of the 10 volunteers (five male and five female) are shown in Fig. 2. The first column of each subject represents the photograph of the target imaging region and the remaining columns represent the obtained ear vascular MAP images according to the imaging area. Upon analyzing the majority of participants' vasculature, it was observed that vessels were distributed fairly evenly across a range of diameters, from larger to smaller. Overall, the vascular distribution was extracted well from the three selected regions from all the volunteers. It was also possible to discriminate the volunteer signals because of the different biological characteristics and curvature of each studied ear.

Specifically, volunteers 4 and 5 commonly had fewer blood vessels than others, however, it was possible to distinguish them based on the fact that volunteer 5 had larger diameter blood vessels in region C than those of volunteer 4. In addition, volunteer 7 benefited from thin skin that allowed the clear distinction of her vascular distribution, resulted in higher signal-to-noise ratio (SNR) values and penetration depth. In the case of volunteer 9, the curvature of the ear affected the intensities of the OCTA signals in areas A and C, yet the overall distribution of micro vessels was discernible. Based on these results, when comparing the vascular MAP images of the 10 volunteers, the quality of the images obtained through vascular angiography was influenced by factors such as the depth of light penetration, SNR, and system robustness. Although variations in vascular distribution and density occur owing to volunteers' biological characteristics, such as skin condition, body fat percentage, and motion artifacts, the proposed OCTA-based ear vasculature imaging was able to observe the microvessel map in different conditions.

### Quantitative analysis of vascular distribution

**FIG. 3.**
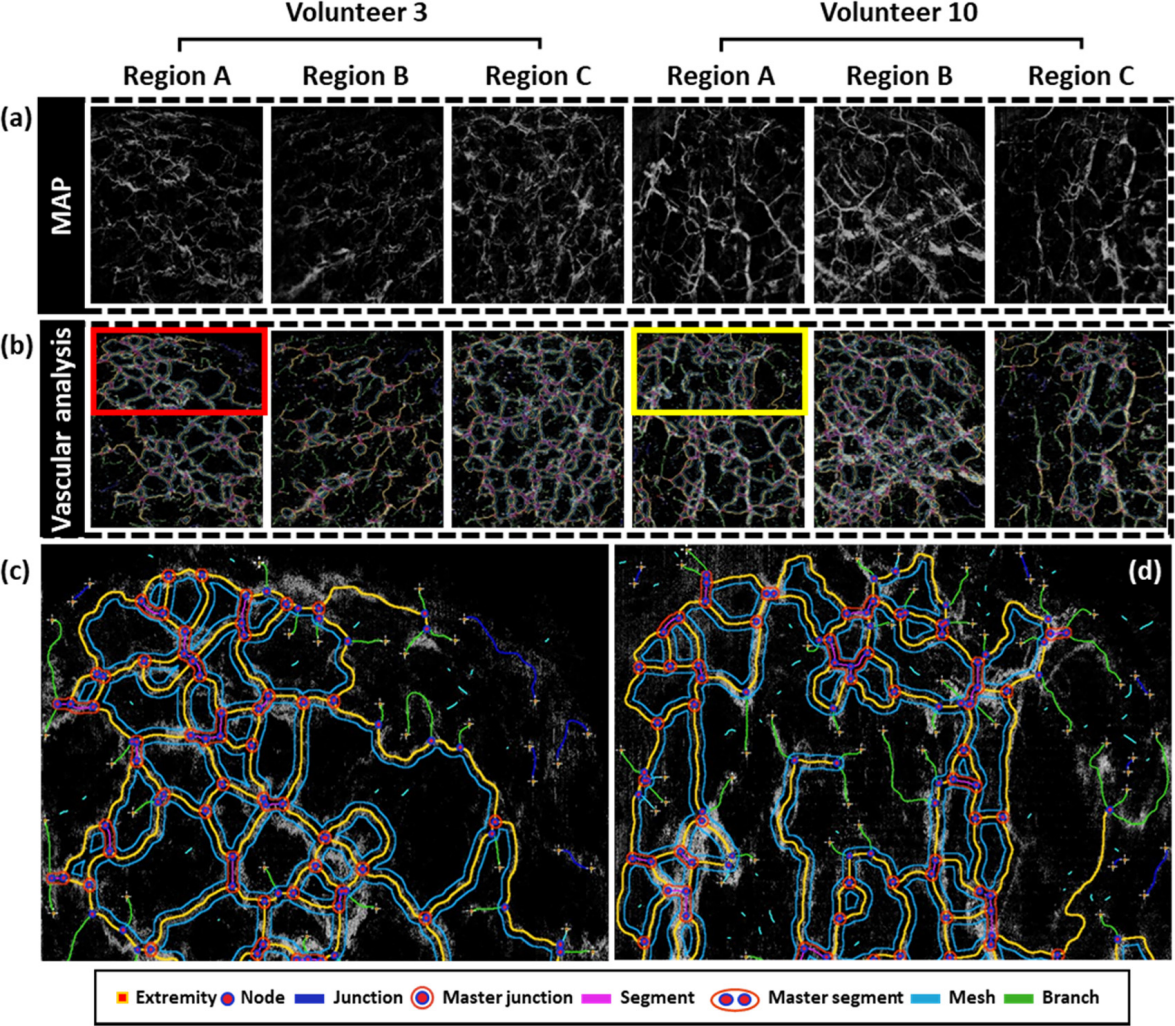
Maximum amplitude projection (MAP) images of the participants and angiogenesis analysis results. (a) MAP images of volunteer 3 and volunteer 10. (b) Vascular analysis of images of volunteer 3 and volunteer 10. (c) and (d) An enlarged image of the vascular analysis in region A of volunteer 3 and volunteer 10.

To analyze quantitatively the characteristics of the ear vasculature for each individual, we used map images, which were converted to grayscale and subjected to data processing by applying a threshold to reduce noise and highlight only the blood vessels. The first row of Fig. 3(a) shows the representative transformed map images of regions A, B, and C of volunteer 3 and volunteer 10. Subsequently, we utilized a software tool called Vascular Genesis Analyzer, based on Java and built upon the image processing software ImageJ.^40^ This analyzer was designed to analyze the vascular network images with 20 different variables for deep and quantitative analysis of vascular-related assessments. Therefore, we quantified values for various vascular structural parameters and employed them to characterize the features of volunteers using the processed OCTA-based ear vascular MAP as an input; the analyzed results are depicted in the Fig. 3(b). In addition, to emphasize the analyzed results, we showed the zoomed-in images of each region [red and yellow box in Fig. 3(b)] in Figs. 3(c) and 3(d), respectively.

After conducting vascular analysis for all volunteers, quantitative values for 20 variables (introduced in Table I) were obtained. Herein, segments refer to elements delimited by two junctions, branches refer to elements delimited by a junction and one extremity, twigs are branches smaller than a user-defined threshold value, and isolated elements are binary lines that do not branch. Master segments consist of pieces of a tree delimited by two junctions not exclusively associated with one branch, known as master junctions. Master junctions are junctions linking at least three master segments. Optionally, two closely located master junctions can be fused into a unique master junction and meshes are areas enclosed by segments or master segments.

**TABLE I.** Various vascular-related factors used for quantitative analyses.

Denotation	Description	Denotation	Description
a	Number (No.) of extremities	k	No. of branches
b	No. of nodes	l	No. of isolated elements
c	No. of junctions	m	(Total length) - Sum of the lengths of segments, isolated elements, and branches
d	No. of master junctions	n	(Total branching length) - Sum of the lengths of the trees composed of segments and branches
e	No. of master segments	o	Sum of the lengths of the segments
f	Sum of the length of the detected master segments	p	Sum of the lengths of the branches
g	No. of meshes	q	Sum of the lengths of the isolated elements
h	Sum of mesh areas	r	(Branching interval) - Mean distance separating two branches in the trees
i	(No. of pieces) - Sum of the number of segments, isolated elements, and branches	s	(Mesh index) - Mean distance separating two master junctions in the trees
j	No. of segments	t	Mean mesh size

After obtaining the values for the 20 variables, the mean and standard deviation of the variable values were calculated for regions a, b, and c of each volunteer. In Table II, the largest values for each factor among the volunteers are represented in blue, while the smallest values are shown in red. Notably, volunteer 2 yielded the highest values in the cases of four factors (number of branches, total length, total branching length, sum of the length of the branches), whereas volunteer 4 yielded the lowest values in the cases of twelve factors (number of junctions, number of master junctions, number of master segments, sum of the length of the detected master segments, number of meshes, number of pieces, number of segments, number of branches, total length, total branching length, sum of the length of the segments, branching interval). Additionally, volunteer 8 yielded the lowest values in the cases of the number of isolated elements, sum of the length of the isolated elements, and mesh index, while volunteer 10 yielded the highest values in the number of nodes, junctions, master junctions, master segments, sum of the length of the detected master segments, number of meshes, pieces, segments, sum of the length of the segments, and mean mesh size. These individual parameter values for each volunteer allowed for a comparative analysis of vascular variables.

**TABLE II.** Characterized parameters of the ear blood vessels of all the studied volunteers.

Variables	a	b	c	d	e	f	G	h	i	j
Volunteer 1	127.33 ± 11.02	652.67 ± 167.36	185.67 ± 48.21	64.67 ± 24.01	124 ± 52.05	11 034 ± 2856.55	54.67 ± 31.39	369 053.3 ± 144 243.7	388 ± 67.76	226.67 ± 78.95
Volunteer 2	124 ± 9.17	793 ± 155.2	279.33 ± 43.02	87 ± 20.81	161.67 ± 43.89	13 595.33 ± 2114.28	73.33 ± 22.81	483 463 ± 7172471.9	392.33 ± 56.2	280.67 ± 62.23
Volunteer 3	118.67 ± 20.01	725 ± 236.68	207 ± 64.01	80.33 ± 30.09	150.67 ± 65.5	12 890 ± 3764.02	66.33 ± 39.55	478 957.7 ± 328 054.4	371.33 ± 96.57	264.67 ± 102.29
Volunteer 4	118.67 ± 26.35	317.33 ± 207.02	89 ± 59.35	32.33 ± 25.42	54.67 ± 47.72	5628 ± 4324.7	22 ± 20.66	152 484 ± 193685	164.33 ± 90.36	101.33 ± 81.75
Volunteer 5	116.33 ± 19.86	349.67 ± 133.87	102.33 ± 38.14	36.33 ± 13.5	62.67 ± 27.01	6738.33 ± 2221.06	22.33 ± 11.93	736 959.67 ± 276 43.37	208 ± 58.13	114 ± 45.18
Volunteer 6	110 ± 7.94	111 ± 118.83	111 ± 37	36 ± 12.17	65.67 ± 27.59	6762.67 ± 1867.58	29 ± 16.37	140 550.7 ± 111 192.9	222.67 ± 25.17	129.67 ± 57.4
Volunteer 7	89.33 ± 7.09	716 ± 237.78	203 ± 78	85.67 ± 30.29	162 ± 64.16	13 259 ± 4363.18	73.67 ± 33.86	566 384.3 ± 257 617.4	347 ± 117.78	267.33 ± 109.6
Volunteer 8	109.33 ± 7.37	755.67 ± 126.82	221.67 ± 37.74	82.33 ± 13.2	159.33 ± 30.66	119 62.67 ± 1557.69	74.33 ± 16.44	384 357.3 ± 130 551.3	384.67 ± 61.78	285 ± 57.47
Volunteer 9	125.33 ± 10.69	375 ± 46.18	107.67 ± 16.17	41 ± 8.89	67 ± 15.39	6388 ± 1385.41	24.33 ± 6.51	116 200.3 ± 610 31.13	222.67 ± 20.65	123 ± 22.87
Volunteer 10	107.67 ± 20.84	832 ± 277.73	832 ± 227.73	90.33 ± 34.03	173 ± 79.3	139 57.33 ± 4066.96	82 ± 48.51	543 366.3 ± 201 674.3	400.33 ± 113.01	301.67 ± 129

Variables	k	l	m	n	o	p	q	r	s	t
Volunteer 1	96.67 ± 12.7	14.67 ± 2.52	17 286 ± 2454.77	16 470 ± 2651.97	10 741 ± 3277.43	5279 ± 632.9	816 ± 223.38	115.68 ± 52.62	175.63 ± 22.01	7377.5 ± 2276.36
Volunteer 2	1100 ± 4	11.67 ± 1724.75	199 79.33 ± 1724.75	193 25.67 ± 1798.89	133 41.33 ± 2316.28	5984.33 ± 834.36	653.67 ± 90.98	134.09 ± 27.51	158.8 ± 15.89	6537.33 ± 652.72
Volunteer 3	96 ± 19	10.67 ± 236.68	184 51.67 ± 2787.66	17 811 ± 2940.19	12 755.67 ± 1456.16	5055.33 ± 1476.22	640.67 ± 191.19	138.71 ± 55.23	164.95 ± 20.03	6709.73 ± 1955.36
Volunteer 4	69.33 ± 16.5	23.67 ± 11.59	11 779 ± 4243.04	10 013 ± 4721.44	5323.67 ± 4216.92	4689.33 ± 679.05	1766 ± 862.53	72.33 ± 43.86	197.44 ± 85.17	5443.63 ± 5203.14
Volunteer 5	72.33 ± 25.38	21.7 ± 10.5	12 952 ± 3040.05	11 483 ± 3538.22	6312.67 ± 2014.07	5170.33 ± 1614.2	1469 ± 790.5	88.33 ± 9.36	188.13 ± 30.89	3470.2 ± 485.07
Volunteer 6	77.33 ± 4.04	15.67 ± 2.31	130 39.33 ± 1748.63	116 70.67 ± 1847.74	6473 ± 2034.5	5197.67 ± 282.83	1368.67 ± 386.93	84.57 ± 29.6	190.78 ± 27.11	4412.7 ± 1162.12
Volunteer 7	70.07 ± 17.62	9 ± 7.81	177 04.33 ± 5025.54	170 09.67 ± 5650.78	13 237 ± 4685.03	3772.67 ± 987.78	694.67 ± 369.75	184.28 ± 35.93	156.02 ± 7.73	778.07 ± 532.96
Volunteer 8	92 ± 7.55	7.67 ± 3.06	178 17.33 ± 1552.54	174 52.33 ± 1649.17	119 76.67 ± 1742.1	5475.67 ± 288.23	365 ± 159.36	130.02 ± 12.95	145.98 ± 9.15	5143.7 ± 1413.17
Volunteer 9	76.33 ± 8.39	23.33 ± 8.23	120 43.33 ± 1390.1	105 05.33 ± 1977.63	5975.33 ± 1350.85	45.0 ± 994.75	1538 ± 821.93	78.1 ± 14.67	156.38 ± 15.89	4596.37 ± 1986.01
Volunteer 10	89.67 ± 12.5	9 ± 6.24	194 13.33 ± 3238.1	189 09.67 ± 3395.03	139 66.33 ± 4617.39	4943.33 ± 1223.63	503.67 ± 248.54	162.06 ± 69.68	159.45 ± 20.81	7406.5 ± 1991.2

Finally, to verify the distinguishable effectiveness of OCTA-based ear vascular imaging for personal characteristics, a paired-sample t-test for 20 specialized parameters was conducted using SPSS Statistics to determine if there were significant differences (p < 0.05) in these parameters between the two volunteers. Table III lists the characteristic parameters that exhibited statistically significant differences, providing a quantitative understanding of inter-individual variations of the ear vasculature. As a result, each of the volunteers exhibited vascular characteristics that distinguished them from as few as three to as many as seven individuals. In the case of volunteer 3, who had three distinguishing factors from others, it appeared that he had fewer distinct features, sharing relatively common ear vascular parameters with other individuals. Conversely, volunteers 7 and 8 demonstrated seven distinguishing factors from other volunteers, suggesting unique ear vascular variables that set them apart from the rest. Based on this analysis, a comprehensive quantification of ear vasculature information can be obtained, enabling comparisons among individuals.

**TABLE III.** Comparison of characterized parameters that yielded statistically significant differences (p < 0.05).

Volunteer	1	2	3	4	5	6	7	8	9	10
1	⋯	l	⋯	b, c, f, h, j, n, o, r	h	a, c, f, h, i, j, m, n, o	a	l, q	s	p
2	⋯	⋯	⋯	b, c, d, e, g, i, j	b, c, d, e, f, g, h, i, j, m, n, o, t	d, i, k, n, s	t	a, q	b, c, d, e, f, g, h, i, j, m, n, o, r	⋯
3	⋯	⋯	⋯	k	⋯	q	⋯	⋯	m, n	⋯
4	⋯	⋯	⋯	⋯	⋯	⋯	r	b, c, d, e, g, i, j	⋯	c, f, n, o
5	⋯	⋯	⋯	⋯	⋯	⋯	b, f, n, o, r, t	b, c, d, e, f, g, h, i, j, o	⋯	l
6	⋯	⋯	⋯	⋯	⋯	⋯	a, g, l, q, r	b, c, d, e, f, g, i, j, l, m	⋯	b, c, f, h, i, m, n, o, q
7	⋯	⋯	⋯	⋯	⋯	⋯	⋯	n, o, q	r	⋯
8	⋯	⋯	⋯	⋯	⋯	⋯	⋯	⋯	a, b, c, d, e, f, g, h, i, j, m, n, o, r	⋯
9	⋯	⋯	⋯	⋯	⋯	⋯	⋯	⋯	⋯	k
10	⋯	⋯	⋯	⋯	⋯	⋯	⋯	⋯	⋯	⋯

### OCTA-based blood pulsatile waveform measurements

**FIG. 4.**
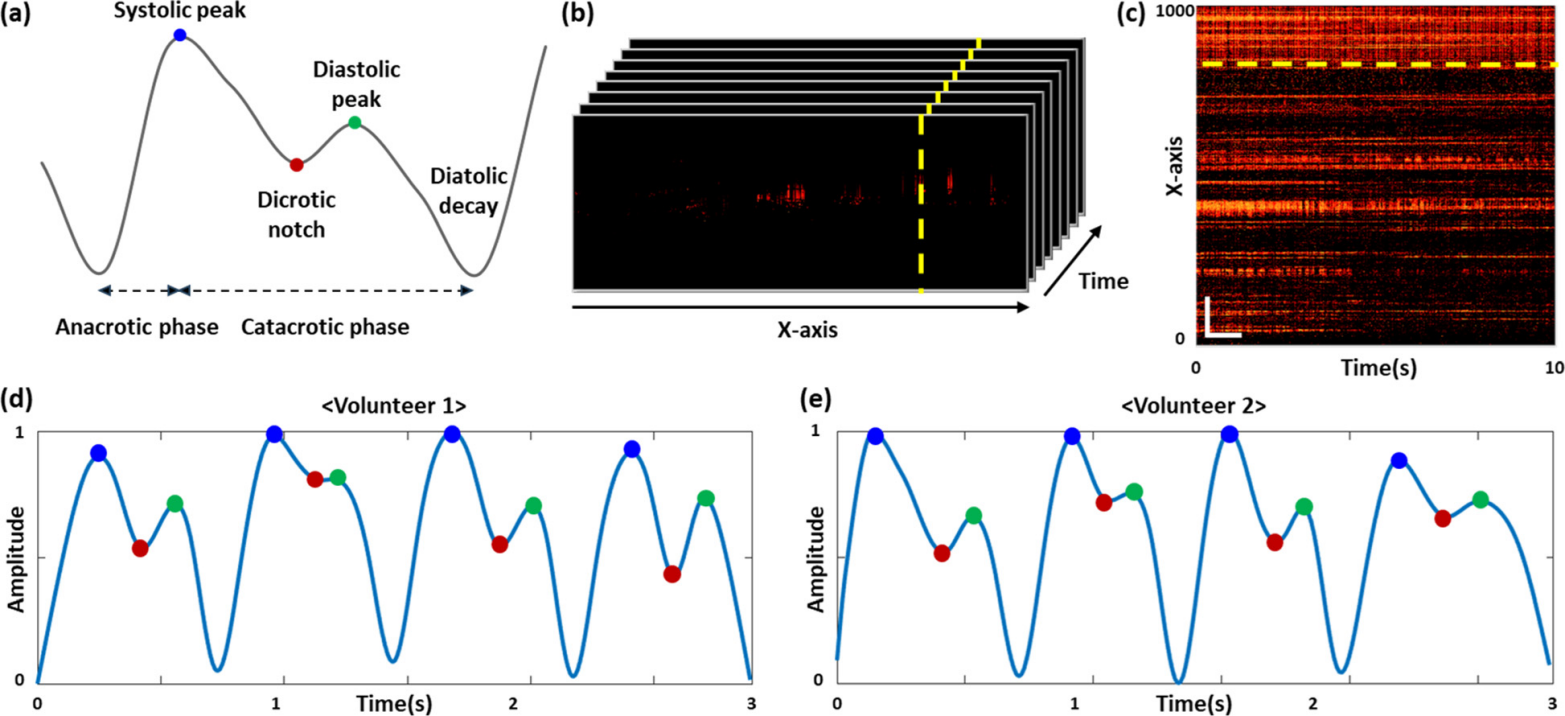
(a) Morphological parameters typically used in PPG signals. (b) Time-series B-scan image showing blood flow variation. (c) Temporal blood flow image extracted repeatedly for 10 s in the area indicated by dotted line in (b). (c) Temporal blood flow image of volunteer 1. (d) and (e) Obtained OCTA pulsatile waveform. Scale bars in (c) are 100 *μ*m (vertical axis) **×** 1 s (horizontal axis).

Figure 4(a) illustrates the typical shape of PPG pulse waves. PPG pulse waves are commonly divided into the anacrotic phase and catacrotic phase, each dominated by systolic ejection and wave reflections, respectively.^41^ Conventionally, there are three distinguished points to compose the PPG signal features: (1) systolic peak (blue dot) (2) dicrotic notch (red dot), and (3) diastolic peak (green dot). Based on this information, we adhered to commonly used PPG principles to characterize the OCTA pulsatile waveform. We randomly imaged a region of the auricle that allows for accurate measurement of blood flow in two volunteers. As shown in Fig. 4(b), we obtained time series of OCTA blood-flow B-scan images at the same position during a 10 s monitoring interval. Each B-scan image consisted of 1000 A-lines; therefore, each B-scan image required 10 ms. In terms of the temporal resolution required to track the blood flow variation, our system speed was 100 frames/s, which was sufficient for precise mapping of the pulsatile signal changes of the volunteers. By repeating 1000 B-scans for 10 s, we were able to map the time-series blood flow signal map at the same position, as shown in Fig. 4(c). We then extracted the blood flow signal fluctuations in conjunction with the time-axis at the signal line [denoted as the yellow-dotted line in Fig. 4(c)] followed by a noise removal process. Consequently, the representative part of the obtained PPG signals from two volunteers are shown in Figs. 4(d) and 4(e). By comparing the typical waveform shape of the PPG signal in Fig. 4(a), the obtained PPG waveforms using the OCTA signal maintained equal mechanism while showing three distinct points. The analysis revealed that it took 2.9 and 2.8 s to obtain four cycles of the waveform, with respective averages of 0.725 and 0.7 s per cycle. The obtained average HR was found to be 82.76 beats per minute (BPM) and 85.71 BPM, thus confirming that it fell within the normal range for adults. Based on these results, the OCTA technique not only provide high-resolution ear vascular maps but also measuring the PPG waveforms simultaneously.

## DISCUSSION

In this study, we demonstrated the OCTA-based noninvasive ear vascular MAP acquisition method and tested 10 subjects using qualitative and quantitative analyses. Our proposed method has the advantage of not only extracting a simple vascular map but also providing comprehensive, intensity-based morphological and depth information for each blood vessel. This capability allows us to offer the information required for the development and application of hearable hardware in a noninvasive and real-time manner, thus making it versatile and applicable in various contexts. In term of the skin color effect on light penetration properties, the resolution and imaging depth of OCT can be affected by absorption. Therefore, it is necessary to consider the melanin content in the skin and its effect on absorption. Although image quality might slightly decrease with increased pigmentation, the differences in image quality and depth among all Fitzpatrick skin types (FSTs) were not significant.^42^ In this study, despite the lower light intensity at this depth, where light intensity at this depth was significantly less in the dark skin than light skin, the imaging is proposed to be qualitatively indiscriminate regarding skin color. The impact of skin color on OCT has been rarely discussed, making it challenging to conclude. Furthermore, there has been research on the impact of the FST, which is recognized as the optimal standard for classifying skin types by dermatologists today, on the transmission of light through the skin.^43^ Despite the decreased light transmission at V–VI skin type according to the reference article, OCT used in our experiment is able to penetrate epidermis and dermis region, which is enough to get the blood vessel signal because of the abundant blood flow at region A, B, and C along with the depth position.

In addition, our ear vascular mapping method can be improved further. First, the architecture of the imaging probe can be modified to adjust the focus and scanning region according to the different curvatures of each region at the ear. Therefore, the various shape of probe tips can enhance the intensity of the OCTA signal and attain consistent image quality regardless of subject conditions. Second, the scanning range of the scanner can be enhanced to cover the entire range of the target location of the SoC sensor once. As the diameter of the designed probe tip limited the scanning range, it can be enlarged by increasing its diameter of probe tip or implementing a dual-sample probe for simultaneous multiple region imaging.^34^ Third, the extractable depth of the vessel signal can be deeper by implementing the multi-focal lens or elongated lens in the sample probe. By modifying the optical configuration of the sample probe, the imaging depth of the OCTA system can be enlarged, which is directly related to the sensitivity and intensity of the obtained blood vessel signal.

## CONCLUSION

In this study, we demonstrated the OCTA-based ear vascular MAP imaging with quantitative analyses to provide comprehensive morphological information for the target location of the SoC sensor. To conduct the experiment to enhance the sensitivity of the OCTA signal, we designed customized a probe tip and integrated it with the sample arm. In terms of the target region of the ear, we acquired OCTA images from three regions that corresponded to areas wherein the posterior auricular artery existed in the external ear, known for its relatively dense vascular distribution. The acquired results of qualitative and quantitative assessments demonstrated the efficacy and superior performance of the proposed OCTA-based ear, vascular MAP acquisition method. Furthermore, the obtained 20 quantitative factors related to vascular distribution information based on vascular analyses enabled quantitative comparisons of their vascular characteristics but also distinguished the volunteers. These results validate the successful acquisition of quantitative information regarding ear vasculature that will aid in the enhancement of the accuracy of PPG sensor measurements, influenced by peripheral blood flow variations. As an aspect of the application of hearable sensor to the real individuals, the feasibility of our proposed method was verified by validating the *ex vivo* experiment-based selected positions of SoC sensor through *in vivo* blood vascular analyzing method using OCTA. Additionally, utilizing the CDV algorithm, we successfully extracted pulsatile blood flow signals using OCTA. Our results revealed that the pulsatile blood flow signals obtained from OCTA share an equal mechanism with PPG signals. Consequently, these study's findings suggest that OCTA has the potential to serve as a noninvasive imaging tool for concurrent measurements of microblood vessel maps with pulsatile waveforms. In conclusion, OCTA-based ear vasculature imaging has a high potential to provide quantitative biological information, including vessel-related factors and biosignals (e.g., PPG). Furthermore, the outcomes of this study can be extensively applied to various applications in dermatology, plastic surgery, and surgical fields, where knowledge of the ear's vascular distribution and blood flow play vital roles.

## METHODS

### Hardware design of OCTA for vascular distribution imaging of the ear

**FIG. 5.**
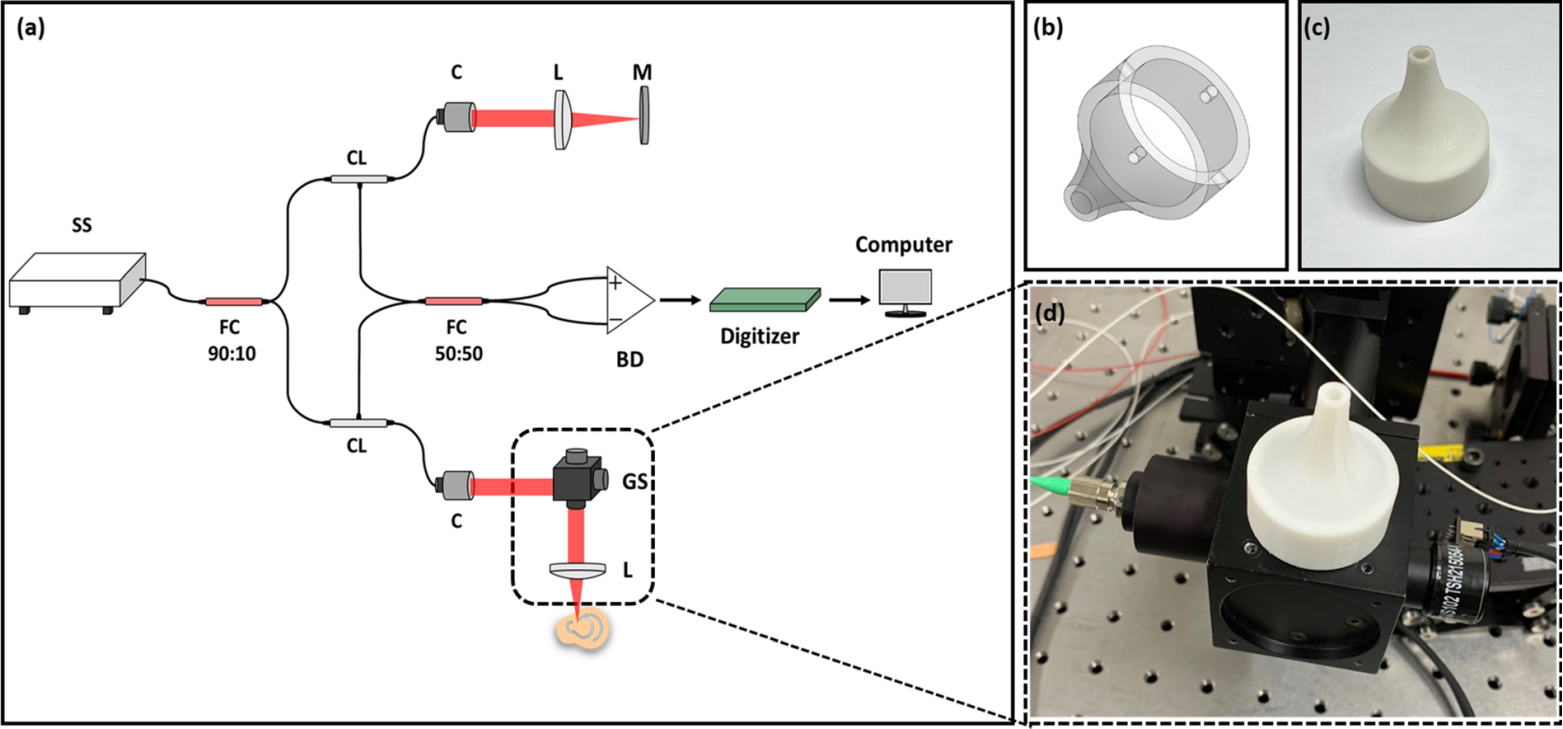
Schematic representation of the used OCTA system. (a) The optical configuration of the proposed system. (b) 3D model of the customized sample probe used for ear imaging. (c) Photograph of the customized sample probe using 3D printing. (d) Photograph of the integrated sample probe in the OCTA system. BD, balanced detector; C, collimator; CL, circulator; FC, fiber coupler; GS, galvanometer scanner; L, lens; M, mirror; and SS, swept source.

The optical configuration of the swept-source OCTA system employed for imaging of the outer ear is depicted in Fig. 5(a). A swept-laser imaging source (SL134051, Thorlabs, USA) with a central wavelength of 1300 nm, a sweep rate of 400 kHz, and an imaging depth range of 3 mm was utilized. The light emitted from the source was connected to a 90:10 fiber coupler (TW1300R2A2, Thorlabs, USA). The distributed light was then directed to the input arm of a circulator (CIR-1310-50-APC, Thorlabs, USA) connected to both the reference and the sample arm. Specifically, the reference arm consisted of a collimator (F260APC-C, Thorlabs, USA), lens (AC254-030-C, Thorlabs, USA), and mirror (PF10-03-P01, Thorlabs, USA), while the sample arm comprised a collimator (F260APC-C, Thorlabs, USA) and a 2-axis galvanometer scanner (GVS002, Thorlabs, USA). The 50:50 ratio coupler (TW1300R5A2, Thorlabs, USA) collected the signal, which was subsequently connected to a balanced detector (PDB480C-AC, Thorlabs, USA). To digitize the photodetector output, a digitizer (ATS9373, Alazar Technologies Inc., Canada) was employed. For accurate OCTA signal acquisitions, it is crucial to image precisely the focused region without artifacts including motion and out-of-focus. Hence, a custom probe tip was meticulously designed using three-dimensional (3D) modeling software and manufactured for this study. The designed probe tip and photograph of the printed tip are illustrated in Figs. 5(b) and 5(c). In terms of the length of the probe tip, which is directly related to the imaging stability and sensitivity of measured OCTA signal, we tested five different types whose lengths varied from 31.3 to 35.3 mm at 1 mm intervals. Based on the experimental tests performed to choose the optimal probe in terms of signal intensity, the 32.3 mm tip exhibited superior imaging performance and was integrated into the sample arm to conduct the experiment, as shown in Fig. 5(d).

### Software flow chart for vascular signal processing

**FIG. 6.**
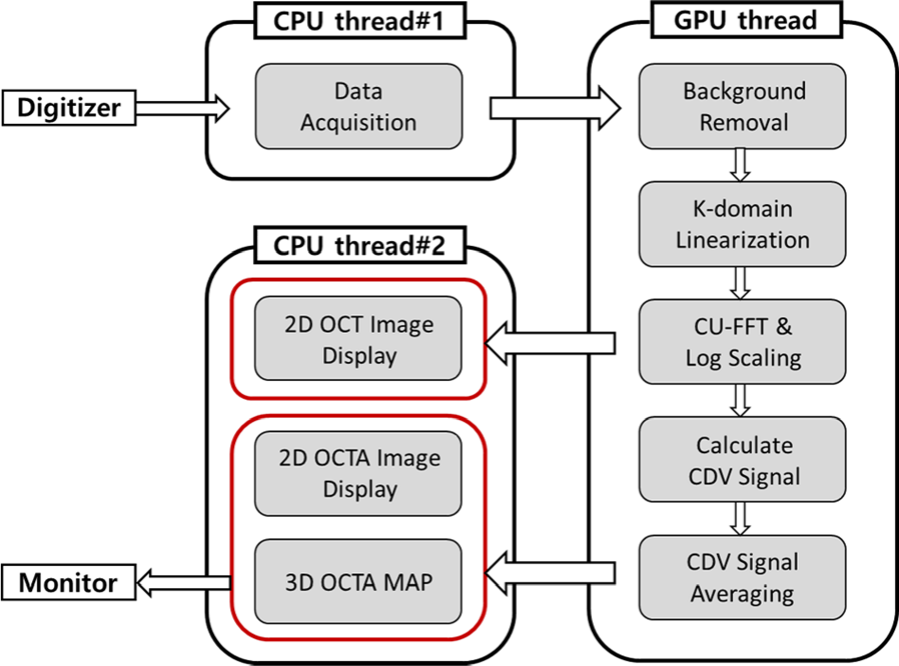
Computer Unified Device Architecthure (CUDA)-based OCTA operating software flow chart used for signal processing.

To process the received interfered signal through the digitizer, we developed LabVIEW-based customized software utilizing graphics processing unit (GPU) technology for real-time imaging and display; the overall flow chart is shown in Fig. 6. The raw OCT signals recorded via the digitizer were sent to the GPU thread for data processing. To visualize the interferometric signals, a series of sequential processing steps were applied: (1) background removal, (2) k-linearization of the full range complex spectrum, (3) Compute Unified Device Architecture (CUDA) fast Fourier transform (CU-FFT), and (4) logarithmic scaling. Based on these processes, the intensity-based cross-sectional OCT images were obtained and ready to display. Furthermore, to extract vascular information at the microscale level, complex differential variance (CDV) analysis was employed to extract vascular information based on the internal signals obtained with OCTA. CDV values were calculated by comparing the signals from multiple scans of the same sample location at different time points.^44^ To address noise-related biases, CDV value pairs from repeated scans were spatially and temporally averaged. For this experiment, four repeated scans were performed to obtain CDV value calculations. The processed data were transferred back to the CPU threads and were subsequently displayed on the monitor as 2D OCT and OCTA images, along with a 3D OCTA MAP. By using our customized software, it became possible to obtain noninvasively the vascular map of the ear and the structural information in real time.

### Target imaging regions and volunteer details

The auricle is supplied by the superficial temporal and the posterior auricular arteries that originate from the external carotid artery.^45^ The posterior auricular artery serves as the dominant blood supply for the auricle.^46^ The anterior surface of the auricle mainly receives blood from the perforating branches of the posterior auricular artery, which traverse the medial aspect of the auricle, including the triangular fossa, conchal bowl, cavum of the concha, helical root, and lobule, and then proceed outward and laterally.^47^ This vasculature network ensures a sufficient arterial blood supply even with a single arterial system.^47^ As shown in Fig. 1(I), the perforating branches of the posterior auricular artery are predominantly situated around the entrance of the external auditory canal, concha cavity, and the cymba concha.^48^ In contrast, in the superficial temporal artery case, the branches primarily originate from regions around the tragus and helical rim, while the formation of branches extending into the concha area is less common.^48^ Consequently, the cymba concha area corresponds to the vascular distribution region of the posterior auricular artery. When performing 3D analyses of the vascular distribution in a part of the external ear obtained from cadavers, it was observed that within the defined external ear region (3 × 5.5 mm^2^), 21.7 microvessels were present (on average) with an average diameter of 71.58 *μ*m.^49^ This accounted for approximately 2.71% of the total area. In our study, we selected three representative regions [A, B, and C in Fig. 1(I)] for the analysis of auricular vascular distribution to determine the location of the targeted SoC sensor where the posterior auricular artery was situated. So we imaged only the right ear because it is anatomically established that there is a rich vascular network in the regions of both ears, and we believed that obtaining average values for the three regions in the right ear alone would suffice.

Additionally, we acquired vascular information from regions A, B, and C from 10 healthy volunteers of various age groups to enable diversified imaging of randomly distributed vessels across different regions. Details of the healthy volunteers are provided in Table IV. Healthy volunteers aged over 25 years with no comorbidities were recruited after seeing an invitation posted on the bulletin board of the electronic engineering department building. Those who expressed interest were sent an invitation letter by the study researcher, which included a volunteer information sheet and a consent form. If the volunteers met the eligibility criteria and provided consent, they were contacted to schedule an imaging date via message. Prior to the imaging session, all volunteers signed an informed consent form. During the imaging trials, an otolaryngologist supervised the process. Participants were briefed on the experiment procedure and then seated in a reclining chair for imaging. Using a handheld OCTA probe, the study researcher conducted ear vascular map imaging in three different positions.

**TABLE IV.** Personal information of healthy volunteers.

Label	Detail information	Label	Detail information
Volunteer 1	28 years old, 179 cm, 82 kg	Volunteer 6	36 years old, 169 cm, 48 kg
Volunteer 2	26 years old, 171 cm, 78 kg	Volunteer 7	49 years old, 170 cm, 58 kg
Volunteer 3	32 years old, 180 cm, 80 kg	Volunteer 8	25 years old, 159 cm, 51 kg
Volunteer 4	30 years old, 175 cm, 90 kg	Volunteer 9	24 years old, 161 cm, 50 kg
Volunteer 5	39 years old, 175 cm, 68 kg	Volunteer 10	46 years old, 167 cm, 57 kg

## Data Availability

The data that support the findings of this study are available from the corresponding authors upon reasonable request.
